# Lightweight and Real-Time Infrared Image Processor Based on FPGA

**DOI:** 10.3390/s24041333

**Published:** 2024-02-19

**Authors:** Xiaoqing Wang, Xiang He, Xiangyu Zhu, Fu Zheng, Jingqi Zhang

**Affiliations:** 1 Center for Quantum Technology Research and Key Laboratory of Advanced Optoelectronic Quantum Architecture and Measurements (MOE), School of Physics, Beijing Institute of Technology, Beijing 100081, China; 2 Key Laboratory of Electronics and Information Technology for Space Systems, National Space Science Center, Chinese Academy of Sciences, Beijing 100090, China; 3 School of Integrated Circuits and Electronics, Beijing Institute of Technology, Beijing 100081, China

**Keywords:** infrared image processing, field programmable gate array, non-uniformity correction, edge-preserving filtering

## Abstract

This paper presents an FPGA-based lightweight and real-time infrared image processor based on a series of hardware-oriented lightweight algorithms. The two-point correction algorithm based on blackbody radiation is introduced to calibrate the non-uniformity of the sensor. With precomputed gain and offset matrices, the design can achieve real-time non-uniformity correction with a resolution of 640×480. The blind pixel detection algorithm employs the first-level approximation to simplify multiple iterative computations. The blind pixel compensation algorithm in our design is constructed on the side-window-filtering method. The results of eight convolution kernels for side windows are computed simultaneously to improve the processing speed. Due to the proposed side-window-filtering-based blind pixel compensation algorithm, blind pixels can be effectively compensated while details in the image are preserved. Before image output, we also incorporated lightweight histogram equalization to make the processed image more easily observable to the human eyes. The proposed lightweight infrared image processor is implemented on Xilinx XC7A100T-2. Our proposed lightweight infrared image processor costs 10,894 LUTs, 9367 FFs, 4 BRAMs, and 5 DSP48. Under a 50 MHz clock, the processor achieves a speed of 30 frames per second at the cost of 1800 mW. The maximum operating frequency of our proposed processor can reach 186 MHz. Compared with existing similar works, our proposed infrared image processor incurs minimal resource overhead and has lower power consumption.

## 1. Introduction

### 1.1. Background

Infrared (IR) imaging technology has emerged as a cornerstone in various fields, including but not limited to surveillance, medical diagnostics, environmental monitoring, military and industrial applications [1,2,3,4]. Infrared sensors can absorb the incident IR radiant flux and convert it to electrical signals [5]. The ability of infrared imaging to capture thermal radiation invisible to the human eye has rendered it indispensable in scenarios requiring temperature measurements, object detection in low-light conditions, and anomaly detection [6,7]. However, the computational demands imposed by infrared image processing algorithms are rather large, presenting a significant bottleneck in achieving real-time performance, energy efficiency, and portability [8,9].

Currently, due to limitations in the resolution, signal-to-noise ratio, and other performance factors of infrared detectors caused by both physical and technical constraints, infrared images typically exhibit characteristics such as high noise, low contrast, significant non-uniformity, and loss of detail [10]. Moreover, infrared imaging systems require real-time processing and feedback, especially in practical applications like infrared target tracking [11]. Conventional computing platforms face challenges when processing infrared image data due to the intricacies of the algorithms involved, resulting in limitations regarding computational complexity, power consumption, and portability [12]. To address these challenges, there has been a growing trend to deploy infrared image processing algorithms on the field-programmable gate arrays (FPGAs) platforms to enhance the imaging quality of infrared systems [13,14].

FPGA is a reconfigurable hardware platform that enables parallel computation, flexible configuration and optimization, and reduction of memory access latency and transfer overhead for infrared image processing. This improves the speed, efficiency, accuracy, stability, and real-time performance of infrared image processing [15].

### 1.2. Related Works

Inostroza [16] presented an embedded real-time system architecture on FPGA that implements multimodal registration to enable dual-camera spatiotemporal feature extraction in a skin cancer screening application. The design runs at 540 frames per second (FPS) with a 135 MHz clock and consumes 1.8 W. Rong [17] proposed an improved neural-network-based non-uniformity correction (NUC) algorithm by the guided image filter and the projection-based motion detection algorithm. An FPGA-based hardware design was also introduced to realize the proposed NUC algorithm at the cost of 19,596 combinational adaptive look-up tables (ALUTs) and 20,118 dedicated logic registers. The proposed design can achieve a frame rate of more than 180 FPS. Redlich [18] presented a digital fixed-point FPGA architecture that performs real-time non-uniformity correction using the constant range algorithm. The architecture was implemented on XC6SLX45T with a resolution of 640×480 14-bit pixels at up to 238 FPS with low resource utilization and adds only 13 mW to the FPGA power. Njuguna [19] proposed an FPGA architecture for two-point non-uniformity correction and bad pixel replacement. The design realizes a maximum operating frequency of 300 MHz and consumes 4293 look-up tables (LUTs), 4261 flip-flops, 11 digital signal-processing blocks, and five block random access memories. Lielāmurs [20] proposed an approach, namely blackbody calibration, to perform non-uniformity correction using a two-point method with a resolution of 320×240 at the cost of 2159 slices on XC5VLX110. Sosnowski [21] presented an image processing and analysis system to perform non-uniformity correction and bad pixel mapping. The design was implemented on EP2C35F672, consuming 33,216 logic elements, 483,840 bits of memory, and 35 embedded hardware multipliers. Bieszczad [22] presented a digital processing system design with state-of-the-art SoC-FPGA using a Cortex-A9 processor embedded in Altera FPGA 5CSEMA5F31C6N. The system comprises two detector arrays integrated with continuously rotating polarizers, achieving frame rates that span from 12 to 46 frames per second. Tasu [23] proposed a high-performance compensation algorithm with a redesigned linear conversion architecture to enhance image details through a segmented method and improve compensation. The architecture was implemented on Kintex-7 at the cost of 458 LUTs.

### 1.3. Motivation

In a significant portion of existing research, infrared image processing only serves merely as a step within the broader system without the absolute focus placed on refining the intricacies of infrared image processing. In these studies, the methods employed for infrared image processing typically encompass only the NUC function. However, even after undergoing NUC, the resulting infrared images remain less conducive to direct human observation. Furthermore, another subset of existing research tends to emphasize achieving the highest frame rates in infrared image processing. This is often achieved by simplifying the algorithms, disregarding the fact that the majority of infrared sensors cannot support such elevated output frame rates. Consequently, a significant amount of performance is wasted in practical application scenarios. Additionally, there is a lack of algorithmic designs specifically tailored for human eye observation. Furthermore, many existing studies implement non-edge-preserving (NEP) filtering algorithms, leading to a substantial loss of image details in the processed infrared images. Designs with edge-preserving capabilities are often constrained by complex edge-preserving (EP) filtering algorithms, contributing to a reduction in the overall speed of infrared image processing.

In light of the aforementioned challenges, this paper aims to propose a lightweight infrared image processor tailored for applications where direct human observation is essential. This processor can find widespread application in scenarios such as handheld infrared devices and small unmanned aerial vehicles equipped with infrared observation systems. A departure from the excessive pursuit of the highest processing frame rates for infrared images, the primary focus is on ensuring that the processed infrared images are suitable for direct human observation. Simultaneously, there is an optimization of algorithms and circuit designs to enhance the highest processing frame rates of infrared images, aligning with the maximum output frame rates supported by the majority of infrared sensors. This paper achieves high-definition infrared image processing by adding edge-preserving filtering algorithms to enhance image quality while compromising on the output frame rate and clock frequency of the image to pursue a balanced processing approach. This holistic approach seeks to balance human interpretability with processing efficiency, addressing the limitations and shortcomings prevalent in current infrared image processing research.

### 1.4. Contribution and Paper Structure

By leveraging a lightweight FPGA platform, this research aims to bridge the gap between real-time processing constraints and the preservation of crucial details in infrared imagery. The envisioned infrared image processor seeks to strike a balance between computational efficiency and retaining pertinent image information, catering to the exigencies of real-time processing while enhancing the interpretability of processed infrared images for human observers.

The main contributions in this paper are as follows:

1. We proposed a series of lightweight infrared image processing algorithms for hardware implementation, including the two-point correction algorithm, the approximate blind pixel detection algorithm, the side-window-filtering-based blind pixel compensation algorithm, and a lightweight histogram equalization algorithm.
2. The approximate blind pixel detection algorithm employs the first-level approximation in the calculation process of average response rate and average noise voltage. Therefore, iterative computations in the blind pixel detection are eliminated.
3. The side-window-filtering-based blind pixel compensation algorithm utilizes eight side windows as convolution kernels. The results of all kernels are computed simultaneously. Experimental results demonstrate that our proposed SWF blind pixel compensation algorithm not only effectively compensates for blind pixels but also exhibits strong edge preservation capabilities.
4. The proposed lightweight infrared image processor is implemented on FPGA. Under a 50 MHz clock, the processor achieves a speed of 30 frames per second (FPS) with a resolution of 640×480. Meanwhile, compared with existing similar works, our proposed infrared image processor incurs minimal resource overhead and has lower power consumption.

The rest of this paper is organized as follows. Section 2 presents preliminary information for the concept of non-uniformity and blind pixels. The proposed lightweight infrared image processing algorithm is introduced in Section 3. Section 4 presents the architecture of the proposed lightweight infrared image processor. The experiments and results are shown in Section 5. Comparisons with existing designs are discussed in Section 6. Finally, we conclude the paper in Section 7.

## 2. Preliminary

### 2.1. Non-Uniformity of the Infrared Sensors

The non-uniformity (NU) of the infrared sensors has always been a significant factor affecting the quality of the collected infrared images [24,25]. Ideally, when an infrared sensor captures a scene with uniform thermal distribution, the grayscale values of all pixels in the collected infrared image should remain consistent because each pixel in the infrared focal plane array has an identical response curve [26]. However, in reality, it is challenging to achieve perfect consistency in the characteristic parameters of each pixel in the array, and their response curves cannot be completely uniform. These inconsistencies result in different response values when the pixel array receives uniform infrared radiation, thereby affecting the quality of the collected infrared image. This phenomenon is referred to as the NU of the response in the infrared focal plane array. The factors contributing to NU include the following aspects.

1. The NU of infrared images is primarily caused by inherent variations in the infrared sensor [27]. Factors contributing to NU include manufacturing-related issues such as uneven doping, surface state density variations, and gate oxide layer thickness differences. Pixel size variations due to lithography and etching processes also contribute. Material NUs and differences in device transfer efficiency lead to changes in pixel response, creating NUs in the collected infrared images. Controlling these factors is essential for maintaining image quality.
2. The performance of infrared thermal imaging systems is affected by the NUs introduced by the operational state of the infrared focal plane array devices. Key factors include the ambient and operating temperatures of the FPA as well as the drive signals for the infrared detector and readout circuit [28]. Temperature variations in the FPA device impact its radiometric response, affecting the overall uniformity of the focal plane array. Additionally, changes in drive signals contribute to NUs. This variability is determined by the FPA device’s operational state, exhibiting different characteristics in various imaging systems and environments. During device operation, the flow of charge within the semiconductor introduces 1/f noise, which is believed to be caused by surface currents and affecting the NU of the detection device.
3. In infrared imaging systems, external factors such as changes in the infrared radiation intensity of targets and backgrounds, as well as variations in the background radiation of the optical system, impact the NU of focal plane devices [28]. These NUs are closely linked to the actual external conditions, making prediction and testing challenging during the development of focal plane devices and the design of infrared thermal imaging systems.

### 2.2. Blind Pixels of the Infrared Sensors

The infrared sensors are affected by various factors, including the materials used in manufacturing, the production process, readout circuit noise, and environmental temperature [29,30]. The impact of these factors results in certain pixels exhibiting abnormal responses under different lighting conditions, leading to the presence of bright and dark noise points in the captured infrared images. These pixels with abnormal responses are referred to as “blind pixels”. Blind pixels are further categorized into two types: “dead pixels” and “overheated pixels” [31,32,33].

1. Dead Pixels: These are pixels in the infrared focal plane array that have a response rate lower than the average response rate under the same lighting conditions. In the resulting infrared image, dead pixels appear as black noise points. Essentially, these pixels do not adequately respond to the incoming infrared radiation.
2. Overheated Pixels: In contrast, overheated pixels have a response rate higher than the average under the same lighting conditions. They manifest as white noise points in the infrared image. Overheated pixels exhibit an unusually high sensitivity to incoming infrared radiation.

The presence of dead and overheated pixels introduces artifacts in the infrared images, impacting the overall quality and reliability of the imaging system [34,35]. These issues are significant challenges in the development and optimization of short-wave infrared detectors for various applications.

## 3. Proposed Lightweight Infrared Image Processing Algorithm

### 3.1. Calibration Algorithm

Considering the computation complexity and latency of different radiometric calibration algorithms, this article adopts a two-point correction algorithm based on blackbody radiation calibration [36,37,38]. The NU refers to the ratio of the response voltage of all effective pixels of the sensor to the mean response rate when each pixel of the sensor is in uniform infrared radiation with half saturation. The calculation for NU is shown in the following equation.

(1) $$\left\{\begin{array}{c} NU=\frac{1}{V_{avg}}\sqrt{\frac{1}{M\times N-(d+h)}\sum_{i=1}^{M}\sum_{j=1}^{N}{\left(V_{ij}-V_{avg}\right)}^{2}}\\ V_{avg}=\frac{1}{M\times N-(d+h)}\sum_{i=1}^{M}\sum_{j=1}^{N}V_{ij} \end{array}\right.$$

In this context, *M* and *N* represent the dimensions of the sensor, indicating its length and width, respectively. $V_{ij}$ denotes the actual response value of the pixels located at the *i*-th row and *j*-th column, while $V_{avg}$ signifies the average response of the sensor. Additionally, *d* and *h* indicate the quantities of dead and overheated pixels, respectively.

**Figure 1:**
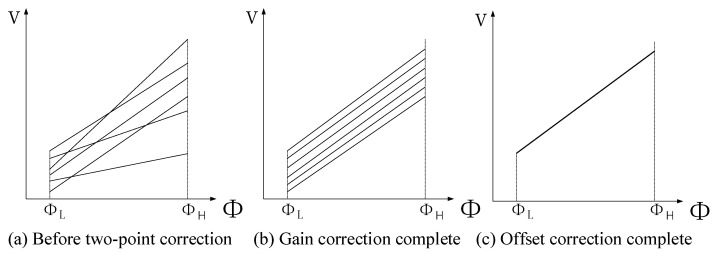
Process of two-point correction.

Although the response function of each pixel unit in the sensor is nonlinear, the response function of the pixel can be approximated as linear within a small working range. Therefore, it is assumed that in a uniform lighting environment, the response value of each pixel in the sensor is linearly related to the radiation flux. So, the response curve of each pixel will form a set of linear clusters with different gain coefficients (*k*) and offsets (*b*). As shown in Figure 1, the vertical axis *V* represents the pixel response, and the horizontal axis ϕ represents irradiation flux. Therefore, the relationship of pixel response $V_{ij}$ and irradiation flux ϕ can be expressed as:

(2) $$V_{ij}=k_{ij}\times \phi +b_{ij}$$

where $k_{ij}$ is the gain coefficient corresponding to the *i*-th row and *j*-th column pixel, and $b_{ij}$ is the offset corresponding to the *i*-th row and *j*-th column pixel.

The two-point correction algorithm aligns the slope of different response functions by rotation and then fits them into one straight line by translation, as illustrated in Figure 1. This algorithm can improve the image quality by making these response functions coincide with the standard response function. High-temperature images and low-temperature images were collected under uniform radiation of high-temperature and low-temperature black bodies, respectively. Thus, the pixel response of the infrared sensor can be obtained as follows:

(3) $$\left\{\begin{array}{c} V_{Lij}=k_{ij}\times \phi_{L}+b_{ij}\\ V_{Hij}=k_{ij}\times \phi_{H}+b_{ij} \end{array}\right.$$

where $V_{Lij}$ is the pixel response of the *i*-th row and *j*-th column pixel in the sensor at low-temperature $\phi_{L}$ radiation flux, and $V_{Hij}$ is the pixel response of the *i*-th row and *j*-th column pixel in the sensor at high-temperature $\phi_{H}$ radiation flux. The average response value of each pixel obtained under varying high and low-temperature conditions is used as the standard response curve for calibrating the infrared sensor. The two-point correction algorithm based on blackbody radiation calibration is shown as follows.

(4) $$P_{Lij}=G_{ij}\times V_{Lij}+O_{ij}$$

(5) $$P_{Hij}=G_{ij}\times V_{Hij}+O_{ij}$$

(6) $$G_{ij}=\frac{\left(P_{Hij}-P_{Lij}\right)}{\left(V_{H}-V_{L}\right)}$$

(7) $$O_{ij}=\frac{\left(P_{Lij}\times V_{Hij}-P_{Hij}\times V_{Lij}\right)}{\left(V_{Hij}-V_{Lij}\right)}$$

where $P_{Lij}$ and $P_{Hij}$ are the pixel response output values corrected at low and high temperatures, respectively. The corrected gain coefficient is $G_{ij}$, and the corrected offset coefficient is $O_{ij}$.

By calculating the above formula, the corrected gain coefficient *G* and the corrected offset coefficient *O* can be obtained. Based on these two sets of coefficient matrices (*G* and *O*), the following collected raw data matrix *V* from the sensor can be corrected to $V_{nuc}$ as shown below.

(8) $$V_{nuc}=G\times V+O$$

### 3.2. Blind Pixel Detection Algorithm

Considering limited hardware resources and computation latency, we propose a lightweight blind pixel detection algorithm based on a fixed blind element judgment standard. The calculation of blind pixel detection involves four parameters: the number of dead pixels *d*, the number of overheated pixels *h*, the average response rate *R*, and the average noise voltage $V_{N}$. During the computation, a mutual correlation exists between the first two parameters (*d* and *h*) and the latter two parameters (*R* and $V_{N}$). In other words, based on the first two parameters, we can derive the latter two parameters, and conversely, we can also determine the first two parameters based on the latter two. Hence, obtaining accurate values necessitates a substantial amount of iterative calculations until all four parameters stabilize within a certain range. This iterative process is time consuming and highly unsuitable for lightweight platforms.

Therefore, we adopted an approximate blind pixel detection algorithm. We first detect blind pixels under the first-level approximation, as shown in the following equation. The average response rate is calculated based on the response of all pixels in the sensor without eliminating dead pixels and overheated pixels.

(9) $$R=\frac{1}{M\times N-d-h}\sum_{i=1}^{M}\sum_{j=1}^{N}r_{\left(i,j\right)}\approx \frac{1}{M\times N}\sum_{i=1}^{M}\sum_{j=1}^{N}r_{\left(i,j\right)}$$

Among them, M×N is the total number of pixels in the image captured by the sensor, and $r_{\left(i,j\right)}$ is the response value of the pixel in the *i*-th row and *j*-th column. We set a fixed criterion at the half of *R*. Then, we mark the pixels that meet the following equation as dead pixels.

(10) $$r_{\left(i,j\right)}-\frac{1}{2}R<0$$

After subtracting the dead image number *d* from the total number of sensor image elements M×N, we calculate the average noise voltage of the remaining pixels. Let $v_{N(i,j)}$ denote the pixel noise voltage in the *i*-th row and *j*-th column of the sensor. The approximation of average noise voltage is calculated by

(11) $$V_{N}=\frac{1}{M\times N-d-h}\sum_{i=1}^{M}\sum_{j=1}^{N}v_{N(i,j)}\approx \frac{1}{M\times N-d}\sum_{i=1}^{M}\sum_{j=1}^{N}v_{N(i,j)}$$

Note that we only eliminate dead pixels from all pixels in this approximate calculation. And we also set a fixed criterion for overheated pixels at the twice of $V_{N}$. Then, we can mark the pixels that meet the following equation as overheated pixels.

(12) $$v_{N(i,j)}-2V_{N}>0$$

### 3.3. Blind Pixel Compensation Algorithm Based on Side Window Filtering

In response to the problem of edge contour blurring in traditional sliding window filtering algorithms, there exist several EP filtering techniques, such as bilateral filtering and guided filtering. However, existing EP filtering algorithms often introduce heightened computational complexity, making them less suitable for real-time hardware processing.

**Figure 2:**
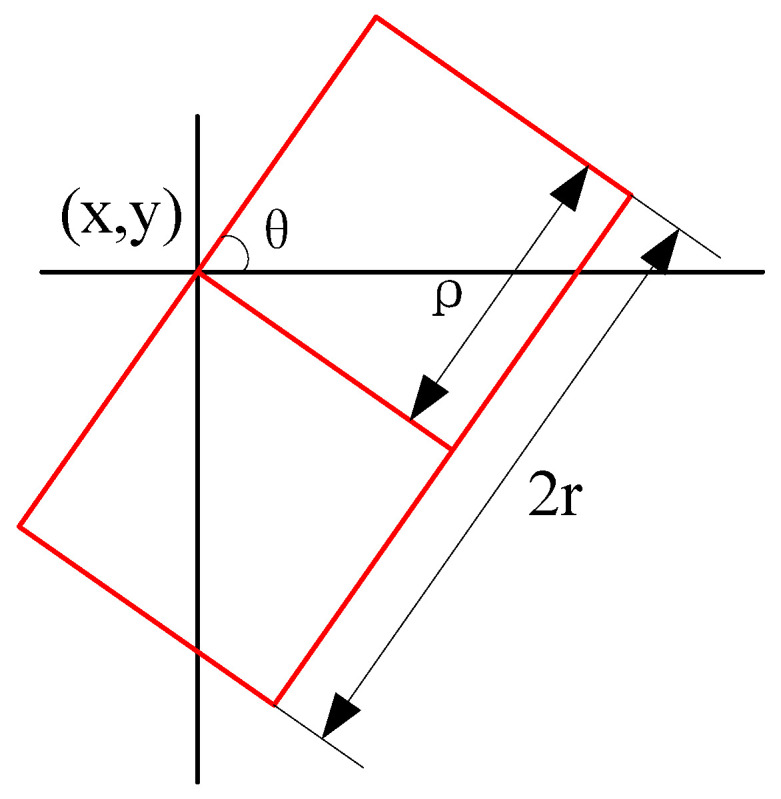
Definition of a side window.

Therefore, this paper proposes an improved side window filtering (SWF) algorithm tailored for real-time hardware implementation. In the SWF algorithm, the pixel to be filtered is placed at the edge rather than the center of the filtering window. Multiple side windows are established to accommodate various filtering scenarios, and the most suitable windows are selected to filter the pixel to be processed. As shown in Figure 2, we define a side window. θ represents the angle between the side window and the horizontal line. 2r denotes the length of the side window, and ρ∈{0,r} is another parameter controlling the size of the side window. (x,y) denotes the location of the side window. By varying θ while keeping (x,y) constant, we can change the orientation of the window, aligning its side with pixel *i*.

**Figure 3:**
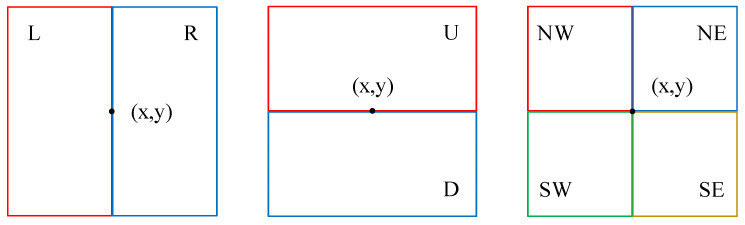
The proposed eight side window types.

From this, it can be observed that for any pixel *i*, the number of its side windows is infinite and depends on the parameter settings. However, in most cases, the chosen parameter settings (e.g., θ is not being multiples of $90^{\circ }$) are not conducive to efficient computation. To simplify computations for discrete pixel points in this design, our objective is only to ensure that the selected side window includes situations where the pixel to be filtered is positioned at the side of the window as well as situations where the pixel is placed at the corner of the window. Therefore, we define eight side windows as shown in Figure 3, which are denoted as Left (*L*), Right (*R*), Up (*U*), Down (*D*), Southwest (SW), Southeast (SE), Northeast (NE), and Northwest (NW). These side windows adequately simulate all scenarios of pixel positions along the edges, where (x,y) represents the coordinates of the pixel to be filtered.

After designing the eight side windows, it is necessary to employ an algorithm to compare and evaluate the filtering effects of all windows to determine the most suitable side window type for the current target pixel. The proposed SWF algorithm utilizes a computational approach depicted in Algorithm 1 to compare and select results from various side windows. The core idea of this algorithm is to utilize the L2 norm to find the filtering result among the eight side windows that are closest to the original pixel value of the target pixel. This approach maximizes the preservation of the original image information in the filtering result. The choice of the filtering kernel function *F* depends on the selected filtering kernel with common options including mean filtering, Gaussian filtering, and median filtering. Given that the algorithm is designed for blind compensation in infrared images, and the characteristics of blind elements are similar to salt-and-pepper noise, both manifesting as alternating bright and dark point noise in the image, the algorithm opts for the use of median filtering as the filtering kernel. This choice is due to its effective handling of salt-and-pepper noise without introducing image blurring issues compared to algorithms such as mean filtering.
**Algorithm 1** Calculate the SWF of each pixel**Require:** *j* denotes the target pixel for filtering, *i* denotes the neighboring pixel of the target pixel, and $\omega_{ij}$ denotes the filtering weight associated with the SWF kernel function *F*, S={L,R,U,D,NW,NE,SW,SE} denotes the set of side window indices.
  1:Compute the result of the target pixel after weighted filtering with different side windows *n*, where $N_{n}$ is the sum of weights of the side window *n*.  2:$I_{n}=\frac{1}{N_{n}}\sum_{j\in \omega_{n}^{n}}\omega_{ij}q_{j}$  3:$N_{n}=\sum_{j\in \omega_{n}^{n}}\omega_{ij},\;n\in S$  4:Determine the optimal side window index based on the Euclidean norm between the filtered pixel values and the target pixel values computed by each side window.  5:$I_{m}=argmin_{n\in S}{∥q_{i}-I_{n}∥}_{2}^{2}$
**Ensure:** $d=I_{m}$


To boost the processing speed of the algorithm above, we employ eight parallel convolution kernels for filtering windows. During the process of traversing the entire image, the results of all eight convolution kernels are computed simultaneously. Meanwhile, the results of the eight convolution kernels are no longer stored to save storage resources. Instead, a hardware-based comparator is utilized to receive the results from eight kernels directly. The comparator will figure out the real-time median filtering result for the target pixel.

### 3.4. Image Enhancement Algorithm

The real-time performance of the hardware implementation of the infrared image processing algorithm is one of the core objectives of this paper. Compared with other image enhancement algorithms, the gray-level statistics and gray-level mapping steps, which have the largest computational complexity of the histogram equalization algorithm, can be performed in parallel based on continuous multi-frame image data, which makes it easier to design the software in parallel and pipeline for the real-time performance of the hardware implementation. Therefore, this paper adopts the image enhancement technique based on histogram equalization to achieve the system function of real-time processing of infrared image enhancement. The specific steps of histogram equalization are as follows:

1. Determine the gray level *L* of the target image. Usually, the gray level value is between 0 and 255.
2. Calculate the proportion $P_{i}$ of the number of pixels $n_{i}$ of each gray level $r_{i}$ in the current image to the total number of pixels *N*;
3. Calculate the cumulative proportion value $S_{i}$ of each gray level from the lowest gray level $r_{0}$ to the current gray level $r_{i}$ in the target image, as shown in Equation (13), where the probability of gray level *L* is 1;
  (13) $$S_{i}=\sum_{j=0}^{i}P\left(r_{j}\right)=\sum_{j=0}^{i}\frac{n_{i}}{N}\;i=0,1,2,\ldots ,L-1$$
4. According to Equation (14), find the pixel mapping relationship, where $r_{i\_new}$ is the new gray level after the current gray level $r_{i}$ is mapped, pix(max) is the preset maximum gray level of the image after equalization, pix(min) is the preset minimum gray level of the image after equalization, and the meaning of the whole formula is to subtract the maximum gray level from the minimum gray level of the image after equalization, multiply by the cumulative probability, add 0.5 and round to an integer;
  (14) $$r_{i\_new}=int\left(\left(pix\left(max\right)-pix\left(min\right)\right)\times S_{i}+0.5\right)$$
5. Grayscale mapping, according to the mapping relationship table obtained by the formula, looks up the table to map the pixels in the original grayscale image to the pixels after histogram equalization.

## 4. Proposed Lightweight Infrared Image Processor Architecture

### 4.1. Overall Architecture

**Figure 4:**
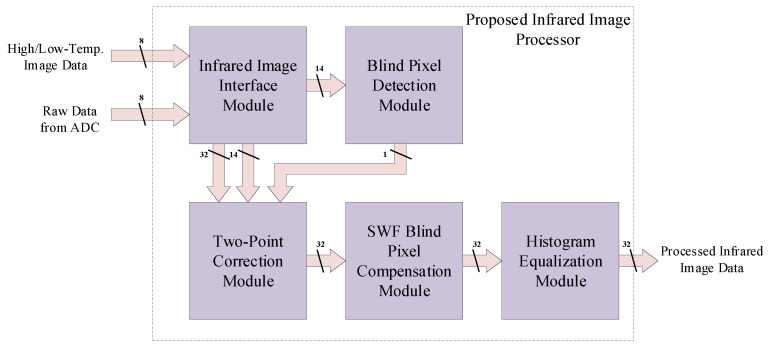
Overall architecture of the proposed lightweight infrared image processor.

The proposed lightweight infrared image processor consists of an infrared image interface module, a two-point correction module, a blind pixel detection module, a blind pixel compensation module, and an image enhancement module. Figure 4 shows the overall hardware architecture of the infrared image processing algorithm unit. To ensure real-time image processing and output, the data throughput of the processor is a crucial performance factor in this design. Therefore, in the processor’s design process, we extensively employed pipeline techniques to enhance throughput and balanced critical path timing to improve the processor’s operating frequency.

**Figure 5:**
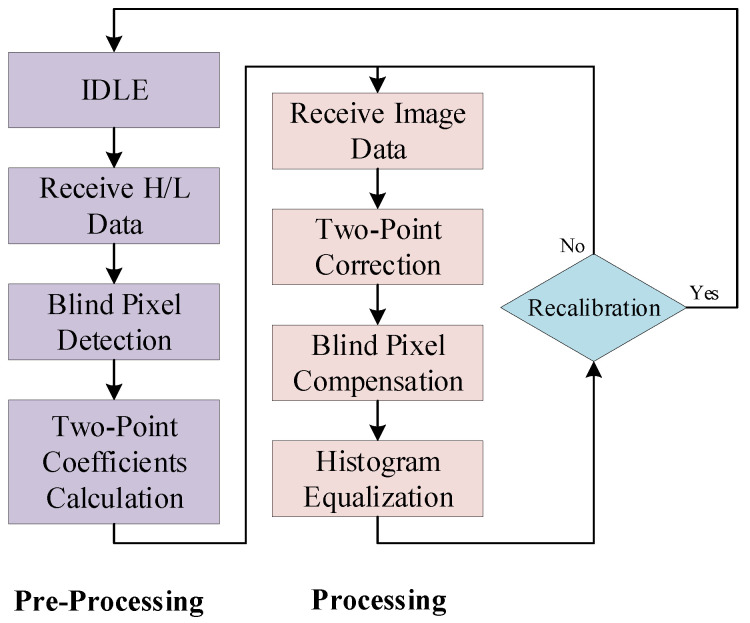
The finite state machine of the proposed lightweight infrared image processor.

The finite state machine (FSM) of the proposed lightweight infrared image processor is illustrated in Figure 5. This design primarily operates in two modes: the pre-processing mode and the normal infrared image processing mode. In the pre-processing mode, which includes four finite state machine states, the control processor acquires raw infrared images at high and low temperatures, calculates two correction matrices, and determines the positions of blind pixels. After completing the pre-processing mode, the processor automatically transitions to the normal infrared image processing mode. Based on the previously calculated correction matrices and blind pixel positions, real-time processing of the infrared image occurs. After blind pixel compensation, the processor performs histogram equalization to enhance the contrast of the infrared image. After processing each frame of the image, the processor needs to determine whether recalibration is necessary. The default logic here is no recalibration is needed, meaning it proceeds to process the next frame of the infrared image immediately. When the recalibration signal is valid, the processor, after completing the current frame processing, re-enters the pre-processing mode.

### 4.2. Infrared Image Interface Module

**Figure 6:**
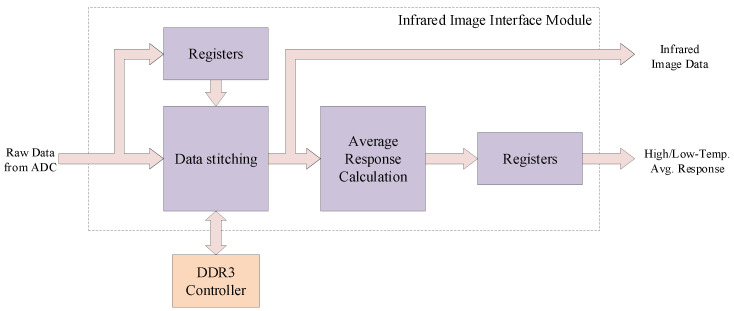
Architecture of infrared image interface module.

The infrared image interface module is responsible for reading image data from the sensor. During the procedure of two-point correction, the infrared image interface module reads high- and low-temperature image data, completes data concatenation, and calculates the average response data for each pixel. Under normal working mode, the infrared image interface module solely performs data concatenating and forwards the stitched data to subsequent modules. The architecture of the infrared image interface module is illustrated in Figure 6. The external input of the raw infrared image data is collected by a 14-bit analog–digital converter (ADC). The actual data for each pixel are formed by concatenating two consecutive bytes. During the two-point correction, the infrared image interface module buffers the concatenated data into DDR3 RAM for use in the calculation process.

In the infrared image interface module, it is essential to define the data format for the processor. As previously mentioned, multiple divisions occur during the process of infrared image processing, involving decimal calculations throughout the entire process. In software-based infrared image processing, the double-precision floating-point format is commonly used for data storage. Although the double-precision floating-point format offers a broad representation range and minimal quantization errors, the computational overhead of double-precision floating-point numbers is substantial, hindering implementation on lightweight hardware platforms. Furthermore, it can reduce data processing speeds, compromising the real-time capability of infrared image processing. To address this, our processor utilizes a custom 32-bit fixed-point number format comprising a 1-bit sign, a 15-bit integer, and a 16-bit fraction. Employing this custom fixed-point number format ensures that the processing speed meets real-time requirements while preserving the precision of processing results as much as possible, thereby achieving better processing effectiveness compared to hardware and software processing results.

### 4.3. Lightweight Two-Point Correction Module

**Figure 7:**
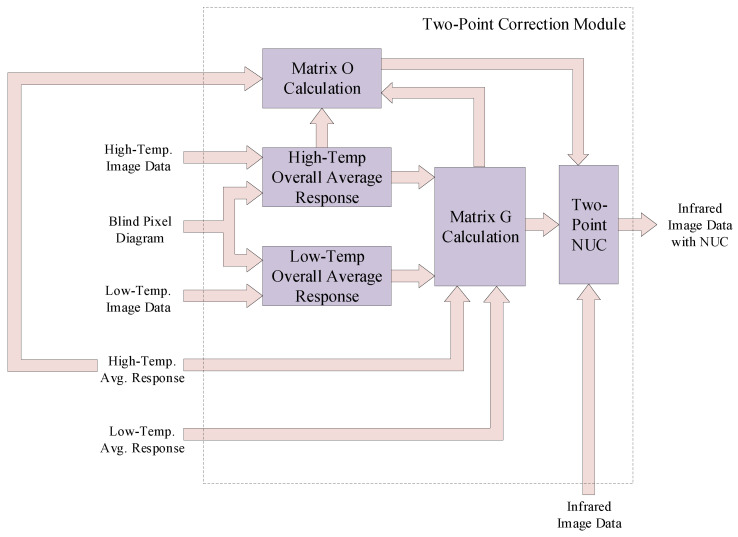
Architecture of lightweight two-point correction module.

The architecture of the lightweight two-point correction module is shown in Figure 7. The input data of the two-point correction module are sourced from both the infrared image interface module and the blind pixel detection module. The infrared image interface module provides high and low-temperature data cached in DDR3 and calculated the average response data for each pixel, and the blind pixel detection module provides position data for blind pixels. Once the infrared image interface module and blind pixel detection module are prepared, the two-point correction module sends data retrieval requests to these modules. Simultaneously, it retrieves position data for blind pixels and high/low-temperature data. Based on the position data for blind pixels, the corresponding data in the high/low-temperature dataset are eliminated. The remaining high/low-temperature data undergo separate accumulations. These accumulations are divided by the count of effective pixels to obtain the overall average response for both high and low temperatures across the entire image.

Once the calculation of the overall average response for high and low temperatures concludes, the two-point correction module continues to send data retrieval requests to the infrared image interface module. Then, it receives the calculated average response data for each pixel concerning high and low temperatures. The two-point correction module computes the difference between the overall average response for high and low temperatures. It also computes the difference between the average response for high and low temperatures for each pixel. These two differences are divided to obtain the corrected gain coefficient matrix.

After the computation of the corrected gain coefficient matrix, the two-point correction module proceeds to calculate the product of the gain coefficient and the average response for high temperatures for each pixel. It then subtracts this product from the overall average response for high temperatures, resulting in the corrected offset coefficient matrix.

Due to division operations involved in the two-point correction module and considering the limited hardware resources on lightweight hardware platforms along with the real-time processing speed requirements, we employed a trial division-based approach to implement lightweight divisions.

Upon input of a dividend and divisor, with the divisor’s bit width *m* as a reference, the comparison is made between the higher *m* bits of the dividend and divisor. If the higher *m* bits of the dividend are greater, the quotient for the corresponding bit is set as one, and the subtraction of the two yields the remainder for this step. Otherwise, if the higher *m* bits of the dividend are not larger, the quotient for the corresponding bit is set as zero, and the higher *m* bits of the dividend directly become the remainder. This process continues, segmenting the dividend until the final division result is obtained. Two shift registers are utilized throughout this procedure: one shift register performs shifting and accumulation for the quotient’s result, while the other handles accumulation and concatenation for the remainder.

### 4.4. Lightweight Blind Pixel Detection Module

**Figure 8:**
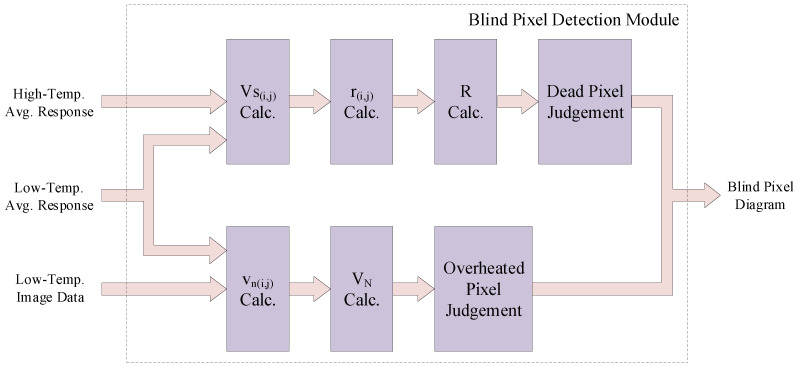
Architecture of lightweight blind pixel detection module.

The architecture of the proposed lightweight blind pixel detection module is illustrated in Figure 8. Utilizing the threshold calibration for dead pixels and overheated pixels outlined in Section 3.2, it identifies the positions of dead pixels and overheated pixels across the entire sensor frame. Align the infrared image sensor with a uniform radiating blackbody, record the response data of each pixel in *K* frames of images at high temperature and low temperature, generate the high and low-temperature pixel response matrices $img_{h}$ and $img_{l}$, then calculate the high and low-temperature averages $favg_{h}$ and $favg_{l}$. Utilize the high and low-temperature averages $favg_{h}$, $favg_{l}$, and the known detector gain coefficient $K_{Gain}$ to calculate the response voltage $V_{s(I,j)}$ of the i-th row and j-th column pixel in the infrared focal plane array. Calculate the pixel voltage response rate $r_{\left(i,j\right)}$ of the *i*-th row and j-th column pixel using the difference in pixel response voltage and known pixel irradiance power factor. Utilize the low-temperature average $favg_{l}$ and the low-temperature pixel response matrix $img_{l}$ to calculate the noise voltage $V_{n}\left(i,j\right)$ of the i-th row and j-th column pixel. Approximate the average voltage response rate *R* and average noise voltage $V_{N}$ obtained from the pixel voltage response rate $r_{\left(i,j\right)}$ and pixel noise voltage $V_{n}\left(i,j\right)$ as initial values for iteration. We progressively compute Equations (9) and (11) for each pixel in a pipelined manner.

The identified positions of dead and overheated pixels across the entire sensor frame are fed into the two-point correction module. This information is utilized to exclude the data corresponding to blind pixels during the statistical process in the two-point correction module. Considering that the quantity of blind pixels across the entire image is significantly lower than that of normal pixels, in our blind pixel calibration process, we assign a value of 1 to denote the blind pixel positions and a value of 0 to denote the positions of normal pixels. This approach aims to reduce the number of logical inversions, thereby lowering the power consumption during processor operation.

**Figure 9:**
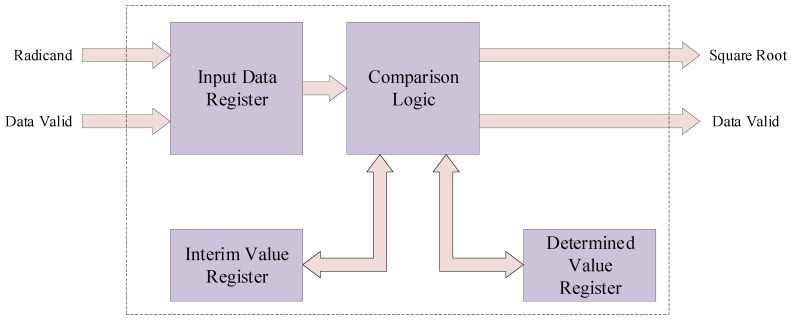
Architecture of proposed square root calculation structure.

The computation process within the blind pixel detection module involves square root operations. Typically, such an operation is implemented by invoking IP cores within FPGA development tools. However, due to the limited hardware resources of this design and the real-time processing speed requirements for infrared imaging, we devised a sequential approximation-based square root calculation structure, as shown in Figure 9. This design concept shares similarities with the previously mentioned trial division approach. Upon inputting the data for square rooting, we initially initialize an interim result.

**Figure 10:**
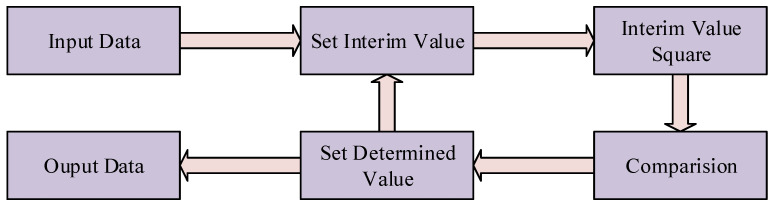
Step of proposed square root calculation structure.

The incremental approximation algorithm is illustrated in Figure 10. First, the data input is denoted as data[n:0]. Then, experimental values $D_{z}\left[n/2:0\right]$ and determined values $D_{q}\left[n/2:0\right]$ are set. Subsequently, in descending order, each bit is successively set to 1 (e.g., setting $D_{z}\left[n/2\right]$ to 1). The square of the experimental value is then compared with the input data. If the square of the experimental value is greater than the input value ($D_{z}^{2}$ > data), the bit is set to 0 (e.g., $D_{q}\left[3\right]$ is set to 0); otherwise ($D_{z}^{2}$ data), the bit is set to 1 (e.g., $D_{q}\left[3\right]$ is set to 1). This iteration is repeated for n/2 times until all levels are compared. After completing all comparisons, the determined value of the last level is output as the square root, and the remainder is obtained as the difference between the input number and the square of the determined value of the last level. Simultaneously, the data valid signal is output, completing the square root operation.

### 4.5. Lightweight SWF Blind Pixel Compensation Module

The input for the SWF blind pixel compensation module is the infrared image processed by the two-point correction module. Although the resulting infrared image is visible to the human eye, its quality is diminished due to the presence of blind pixels. The SWF blind pixel compensation module aims to compensate for these blind pixels in the infrared image while retaining its details.

**Figure 11:**
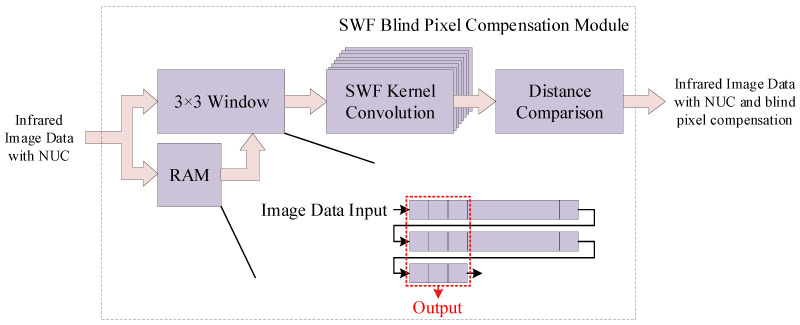
Architecture of proposed SWF blind pixel compensation module.

**Figure 12:**
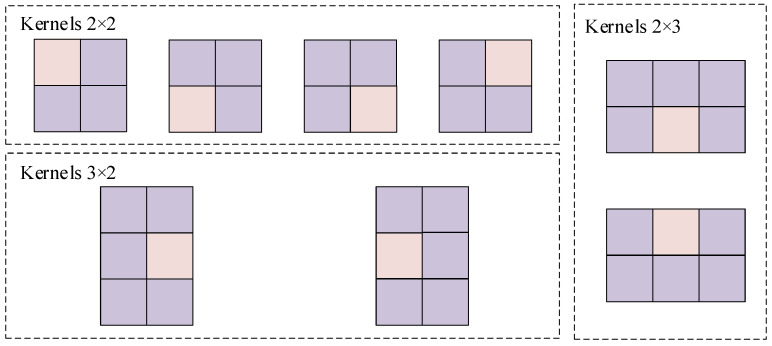
Architecture of proposed SWF kernels.

The architecture of the proposed SWF blind pixel compensation module is shown in Figure 11. Firstly, we determine the side window length for the SWF blind pixel compensation module as 2s+1. Considering the compensation effect for blind pixels, hardware resource consumption, and computational speed, we adopt s=1, thereby setting the side window size to 3×3. Following the output from the two-point correction module into the SWF blind pixel compensation module, we utilize on-chip Block RAMs (BRAMs) to construct a dual-port RAM for data buffering. Given the 3×3 side window size, the space allocated for data buffering includes two complete rows of image pixel data and an additional three pixels. Within the 3×3 buffer space, we construct eight parallel median filtering convolution kernels, as described in Figure 12. In a 3x3 window, we take four 2×2 convolution kernels, two 2×3 convolution kernels, and two 3×2 convolution kernels, each placed at the four corners, midpoints of the top and bottom edges, and midpoints of the left and right edges. All pink-colored blocks in the figure represent the centers of 3×3 windows. Each kernel calculates its convolution result, the median value, while simultaneously computing the distance between each median value and the actual value of the target pixel. Subsequently, we compare the distances from the eight calculations, selecting the kernel with the smallest distance as the output kernel and providing its convolution result as the output. We employ an edge extension method to fill in the outermost circle of pixels. This ensures that pixels located at the boundaries are also processed by the SWF blind pixel compensation module.

### 4.6. Histogram Equalization Module

**Figure 13:**
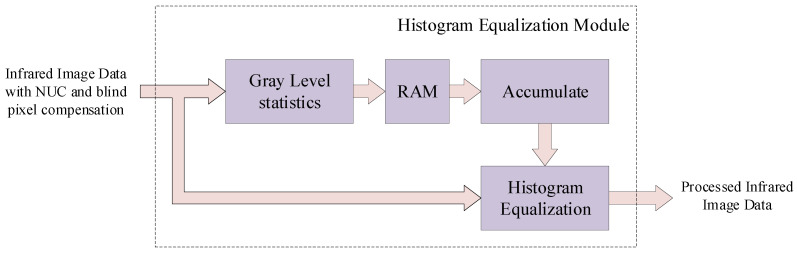
Architecture of proposed histogram equalization module.

**Figure 14:**
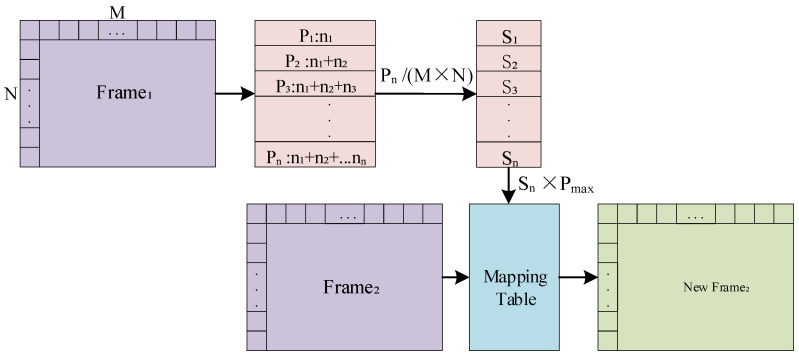
Step of proposed histogram equalization module.

The image enhancement module is the histogram equalization module. This module receives the blind pixel compensated infrared image, counts the gray levels of the image, and then expands and merges the pixels of different gray levels to increase the image contrast and achieve the effect of image enhancement. The histogram equalization module is designed as shown in Figure 13 and the steps of the proposed histogram equalization module is illustrated in Figure 14.

The hardware design of the histogram equalization algorithm adopts a pseudo-equalization design idea. That is, the image of the previous frame is used for statistics, the frame gap is accumulated and normalized, and the current frame is used for normalized mapping output to achieve the purpose of real-time processing. The histogram equalization module includes two parts: a gray-level statistical circuit and a histogram equalization circuit. The histogram equalization module receives the results from the SWF blind pixel compensation module. The results first enter the gray-level statistical circuit. In this procedure, we employ on-chip resources (including BRAMs) to construct dual-port RAM for buffering.

The process of the gray-level statistical circuit is as follows:

1. By traversing the M×N infrared image, we obtain the number of pixels $n_{i}$ corresponding to each gray level and the maximum grayscale value $P_{max}$;
2. Accumulate the number of pixels corresponding to all grayscale values less than this grayscale value, and store the result in RAM.

After the gray level accumulation and statistics of the previous frame image are completed, the image data after blind element compensation of the current frame then enters the histogram equalization circuit. The specific process of hardware implementation of the histogram equalization circuit is as follows:

1. The histogram equalization circuit traverses all pixels in the current frame image in row-first-column order. Each time a pixel is traversed, $P_{n}$ of the current pixel is read out from RAM;
2. Use the built-in divider to divide the readout cumulative sum by the total number of pixels M×N and then multiply it by $P_{max}$ of the current image to complete the normalization operation;
3. Use the normalized calculated gray value to replace the original pixel gray value to complete gray mapping. Until the entire image is replaced, the histogram equalized image is output.

## 5. Experiments and Results

This design includes multiple infrared image processing modules. Therefore, we first simulate each hardware module to ensure its correct function and remarkable effect. Then, we integrate the entire system and test it on the actual FPGA platform.

### 5.1. Algorithm Simulation on FPGA

Considering the lightweight design goal, we selected the ULIS 640 uncooled infrared detector chip as the infrared sensor with a frame resolution of 640×480. At the same time, we use infrared blackbody light sources to provide uniform and stable light sources at high and low temperatures. Under high-temperature and low-temperature conditions, we collected 100 frames of images for correction, respectively.

**Figure 15:**
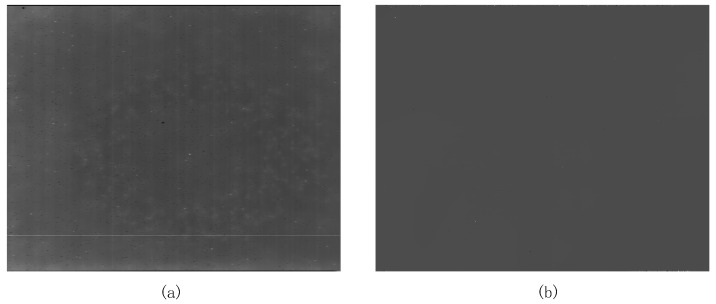
(**a**) The original infrared image; (**b**) the processed image from the two-point correction module.

The original infrared images from the infrared image interface module are shown in Figure 15a. The original image has problems such as blind pixels, noise, horizontal and vertical stripes, etc., that affect the quality of the infrared image. Due to the low image quality of the original infrared image, even if a target appears in the picture at this time, it is very difficult for the human eye to observe. After the processing of the two-point correction module, it can be seen that the quality of the processed image is significantly improved, and the horizontal and vertical stripes are almost invisible to the human eye, as shown in Figure 15b. However, since blind pixel compensation has not yet been performed, there are still bright and dark noises in the image, which are dead pixels.

**Figure 16:**
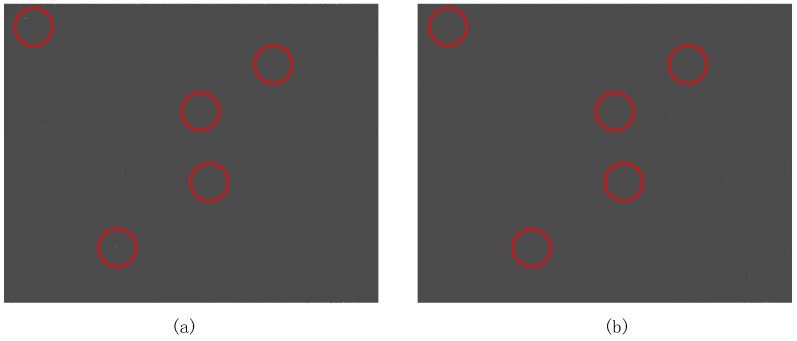
(**a**) The infrared image before blind pixel compensation processing; (**b**) the processed image from the blind element compensation module.

Subsequently, we input the two-point corrected image into the blind element compensation module. We have marked some obvious blind elements in the images with red circles as illustrated in Figure 16. After being processed by the blind element compensation module, the light and dark noise in the image disappears.

**Figure 17:**
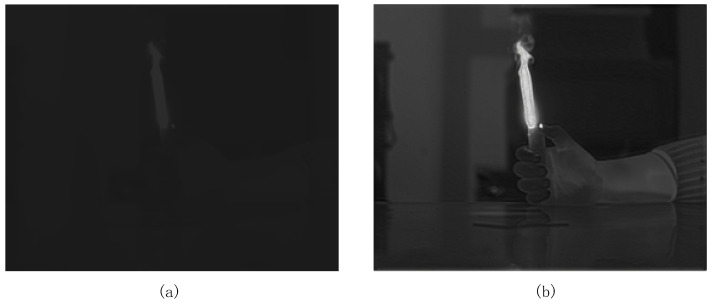
(**a**) The infrared image before histogram equalization processing; (**b**) the processed image from the histogram equalization module.

The following images, seen in Figure 17, show a comparison before and after the Histogram Equalization Module. Before histogram processing, although there are targets in the infrared image, the contrast is very low, making it difficult for the naked eye to observe directly. After processing with the Histogram Equalization Module, not only is the boundary of the flame visible, but other targets with lower temperatures also become clear and distinguishable.

Since the responses of all pixels after two-point correction tend to be consistent and there is no target in the picture, the blind pixel compensation module’s ability to suppress blind pixels and preserve boundaries cannot be well demonstrated. Therefore, we replaced a 256×256 resolution grayscale image with a prominent target and more boundary information to perform an additional simulation on the blind element compensation module. Here, we assess the performance of the blind pixel compensation module using two evaluation metrics: peak signal-to-noise ratio (PSNR) and structural similarity index metric (SSIM).

1. PSNR is a metric commonly used to measure the quality of a reconstructed or compressed signal compared to the original signal. It is often employed in image and video processing to quantify how much the quality of the reconstructed or compressed version deviates from the original, considering both signal fidelity and the presence of noise.
2. SSIM is a metric used to measure the similarity between two images. It is designed to capture perceived changes in structural information, luminance, and contrast that humans often notice.

**Figure 18:**
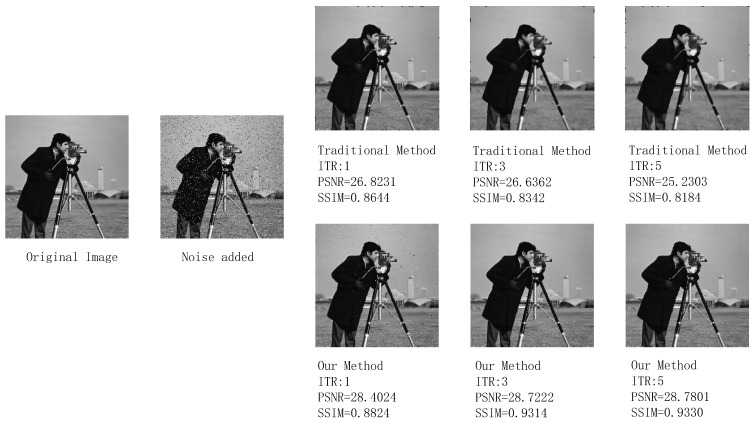
Results after processing with the traditional method and the proposed blind pixel compensation module.

**Figure 19:**
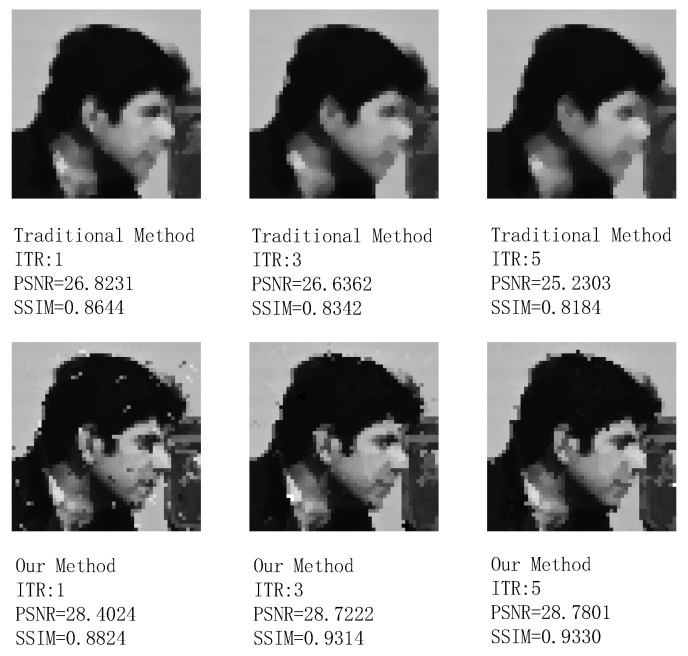
Preservation of image details in the image with the traditional method and the proposed blind pixel compensation module.

The comparison results of the processing effects on grayscale images with artificially added salt-and-pepper noise using traditional median filtering and the improved SWF median filtering algorithm with several times of iteration (ITR) are shown in Figure 18. Figure 19 illustrates the comparison of the processing effects on facial details. Based on PSNR, SSIM, and visual observation, it can be seen that under the same window size and iteration times, the improved SWF median filtering has a significant effect on filtering target edges (such as clothing and hair edges), image boundaries, and the preservation of target details (such as facial information). It also exhibits good filtering performance for dispersed point-like salt-and-pepper noise.

Therefore, although the denoising effect of the improved SWF median filtering is slightly inferior to traditional median filtering, it retains a large amount of image details. Compared to the complete loss of image details caused by retaining a small amount of noise points, this is an unacceptable result in infrared image processing. Hence, we chose improved SWF median filtering over traditional median filtering. Additionally, it can be observed from the figures that a higher number of iterations leads to better image processing results with the improved SWF median filtering algorithm. However, this also increases the hardware resources required, making real-time implementation more challenging. The improvement in image quality after filtering is limited, and a window size of 3×3 is the most commonly used size, striking a balance between resource consumption and filtering effectiveness. Therefore, we choose an iteration time of one and a window size of 3×3 for the improved SWF median filtering.

### 5.2. FPGA Implementation Results

**Figure 20:**
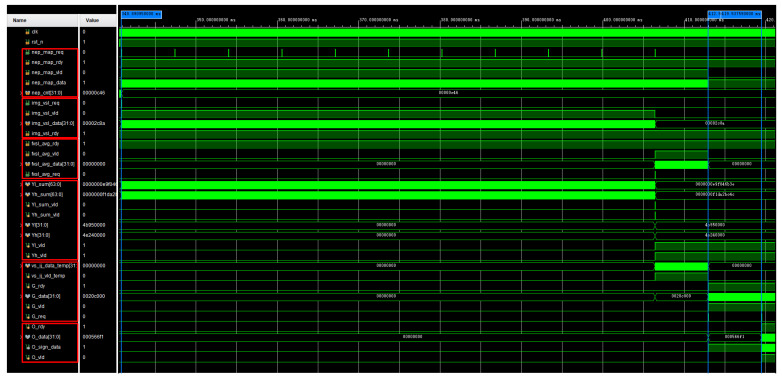
The waveforms correspond to the reading of high and low-temperature data and the precomputation.

After the system is powered on, it first reads the high and low-temperature data and performs subsequent calculations, preparing for the formal image processing. The waveform of the infrared image processor is shown in Figure 20 with a constant frequency of 50 MHz. It can be observed that after the power-on reset, it sequentially completes the reading of high and low-temperature data (img_vsh_rdy, img_vsl_rdy), calculates the mean values of high and low-temperature pixels (fvsh_avg_rdy, fvsl_avg_rdy), and generates the blind pixel diagram (nep_map_rdy). The total time consumed is approximately 340 ms. After this, the processor calculates the corrected gain coefficient matrix and the corrected offset coefficient matrix according to the proposed workflow of the infrared image processor. It can be observed that the total time before the infrared image processor starts processing real-time infrared image data is 419 ms. Afterward, the infrared image processor enters real-time processing mode.

**Figure 21:**
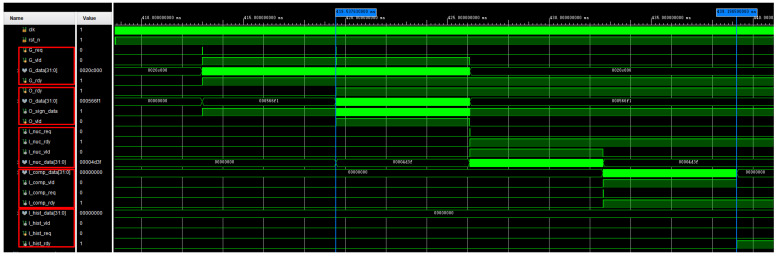
The waveforms correspond to the the real-time processing of infrared images.

The real-time processing section of the infrared image is used to perform non-uniform correction, blind pixel compensation, and image enhancement on the raw infrared image data collected by the infrared detector after the preprocessing part. The amount of data to be processed is significantly reduced compared to the preprocessing part, meeting real-time requirements. The simulated waveform graph is shown in Figure 21. From the graph, it can be observed that under a 50 MHz clock, the entire process, from non-uniform correction and blind pixel compensation to histogram equalization, takes about 20 ms. The target image reading takes approximately 13 ms. Therefore, the real-time processing section of the infrared image processes one frame in about 33 ms, which is equivalent to 30 FPS.

### 5.3. Practical Experiment

**Figure 22:**
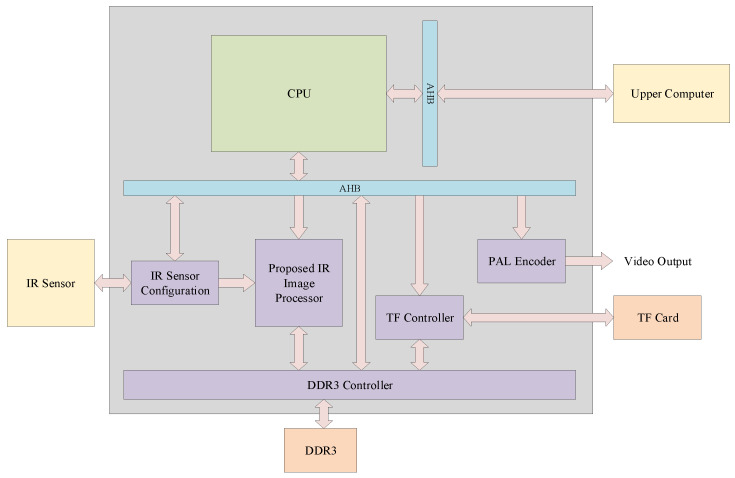
The overall view of the SoC based on the proposed lightweight infrared image processor.

Based on the lightweight infrared image processor proposed in this paper, we constructed a lightweight system-on-chip (SoC) which is shown in Figure 22. The processor in the SoC is based on ARM Cortex-M0 low-power processor, and the bus architecture follows ARM advanced microcontroller bus architecture (AMBA). The architecture of the on-chip system is illustrated in the following diagram.

**Figure 23:**
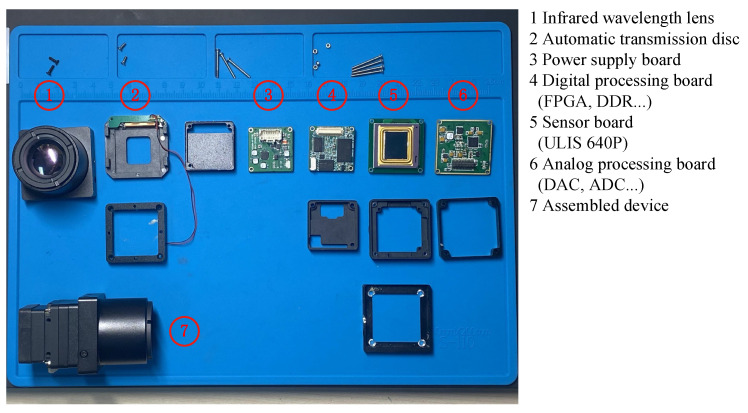
Disassembled infrared observation device and assembled infrared observation device.

After system-level integration, the disassembled structure and assembled device of our handheld infrared observation equipment are shown in Figure 23. The digital processing board carries an FPGA and DDR for building the lightweight infrared image processor proposed in this paper. Our practical experiments are conducted based on this handheld infrared observation device. The dimensions of the device are 67.4 mm × 39.0 mm × 39.0 mm, and it weighs 120 g.

**Figure 24:**
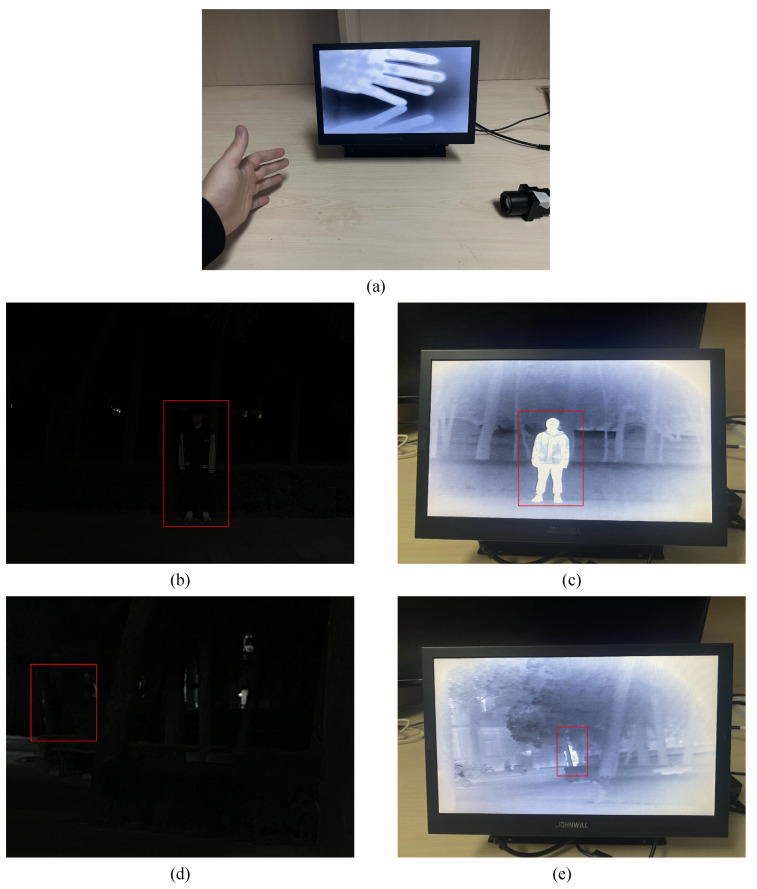
Experiments with the infrared observation device at night. (**a**) The shooting of a human palm in an indoor environment. (**b**) The target (approximately 10 m away) was shot by the visible light camera. (**c**) The target (approximately 10 m away) was shot by the infrared observation device. (**d**) The target (approximately 20 m away) was shot by the visible light camera. (**e**) The target (approximately 20 m away) was shot by the infrared observation device.

The actual experiment of the infrared observation device is shown in Figure 24. Figure 24a shows the shooting of a human palm in an indoor environment. Due to the higher temperature of the palm compared to the ambient temperature, the palm target is very clear, and the boundary is also very sharp. Figure 24b–e compare the shooting conditions of the infrared observation device with a visible light camera in nighttime situations. The target (human) in each picture is labeled with a red box. The image from the visible light camera is captured using an iPhone 13 Pro Max. In close-range situations, with the target (human) approximately 10 m away from the device, the visible light camera can still capture the general outline of the target (human) despite weak ambient light, as seen in Figure 24b. The device, in this case, captures the target (human) clearly with a sharp boundary, as shown in Figure 24c. When the target (human) is about 20 m away from the device, the visible light camera can no longer capture the target, as seen in Figure 24d. However, the infrared observation device can still clearly distinguish the target (human) in the environment, as shown in Figure 24e.

### 5.4. Implementation

This design utilizes the Xilinx Artix-7 low-power FPGA series to construct a lightweight infrared image processor with the specific FPGA model being XC7A100T-2. This FPGA supports DDR3 interface with a transfer speed of up to 1066 Mbps. The development environment for the FPGA is chosen to be Vivado 2019.2. After the synthesis step and the implementation step in Vivado, the implementation results of the overall device are shown in Table 1. The breakdown results for key components and our proposed lightweight infrared image processor are also listed in the table. BRAMs are only employed in the lightweight SWF blind pixel compensation module and the histogram equalization module. The other memories in our design are constructed by FFs.

The expenses of each resource are presented as a percentage of the total on-chip resources in the last row of the table. The overall device consumes 25,515 LUTs, 22,393 FFs, and 8 DSP48. Due to the inclusion of peripherals such as a TF card, DDR3, etc., in this device, the IO resources are utilized to a greater extent, exceeding 50% of the on-chip resources of XC7A100T. Our proposed lightweight infrared image processor costs 10,894 LUTs, 9367 FFs, 4 BRAMs, and 5 DSP48, which are shown in bold.

As for function result, which is shown in Figure 24c,e, we can obtain the location of person by the infrared sensor at night. The red boxes indicate the location of person in Figure 24b,d. As we can see, our sensors can truly obtain the right result. As for timing results, according to the timing report generated by Vivado, our proposed infrared image processor can achieve a maximum operating frequency of 186 MHz. Therefore, it supports a maximum output frame rate of up to 111 FPS. We employed the Xilinx Power Estimator to estimate power consumption. The ambient temperature is set at 60 °C, and power supplies are set as default values. The estimated total on-chip power results of our proposed architecture are 1.812 W@50 MHz and 6.974 W@186 MHz. In the practical experiment, we employed Cortex-M0 to control the *ENABLE* signal for the proposed infrared image processor, and we observed the actual power consumption difference using a stabilized voltage supply. The actual power consumption difference is approximately 1.8 W, which is consistent with the simulation results. However, the sensor in our equipment cannot support an output rate of 111 FPS. Therefore, the power consumption of our device under theoretical limits can only be simulated in Vivado.

## 6. Comparison and Discussion

Our proposed lightweight infrared image processor and existing infrared image processing solutions are summarized in Table 2. First, it should be noted that our infrared image sensor operates at a resolution of 640×480. In the Artix-7 series FPGA, it consumes a total of 10,894 LUTs and 9367 registers. The clock frequency of the image processing module is synthesized to be 186 MHz, resulting in a frame rate of 111FPS. However, due to limitations in the performance of the infrared image sensor, the actual clock frequency is 50 MHz, resulting in an image processing frame rate of 30 FPS. Our work focuses on the implementation of a high-definition infrared image sensor. It can be observed that among all the works, our infrared image sensor has a resolution of 640×480, which is considered high definition for infrared imaging. This also implies that the captured images may contain more noise. This means that compared to small-pixel infrared sensors, we need to consume more hardware resources to process the captured images.

In fact, different FPGA manufacturing processes can lead to variations in performance metrics such as operating frequency and power consumption. Additionally, the manufacturing process can impact the cost of the FPGA. Considering the diverse functional requirements of each design, along with variations in FPGA manufacturing processes and associated costs, providing an entirely fair comparison between these existing designs and ours is challenging. Therefore, in Table 2, we showcase the characteristics and real working performance metrics of each existing design. This information is intended to assist readers in choosing different hardware design approaches, various infrared image processing functionalities (TC: two-point correction, BC: blind pixel compensation, HE: histogram equalization), and FPGAs with different manufacturing processes based on their specific application scenarios.

The architecture, with 500×500 pixels in [16], is designed for multimodal image registration. Therefore, in this design, the requirements for infrared image processing capability are comparatively lower than in our design. Due to the integration of a smaller number of infrared image processing algorithms in [16], the resource overhead of its infrared processing module is minimal, resulting in low power consumption and the ability to achieve a higher frame rate output. However, the primary goal of our design is to focus on the processing of infrared images to achieve higher image quality. Consequently, the integrated infrared image processing algorithms in our design are more comprehensive, leading to higher resource overhead and power consumption. The design in [17] is for the sensor with 256×256 pixels. Therefore, although the design in [17] operates at a frequency similar to that of our design, it supports a higher FPS. The low-power embedded architecture in [18] is a specific design for NUC using the constant range algorithm. Therefore, the hardware resource overhead and power consumption of this design are both at very low levels. Our design, in comparison to [18], encompasses a greater range of functionalities for infrared image processing. The designs [19,20] are similar to our design approach. The functions of NUC and blind pixel compensation are integrated in [19]. For [20], several functions such as geometric transformation, lens distortion correction, and image registration are included. As our design marks the first application of SWF in the field of infrared image processing, its edge-preserving effect allows our algorithm to retain edge information while handling noise. Compared to [19,20], our approach not only eliminates noise but also preserves image clarity to a greater extent. Additionally, in our design, histogram equalization is applied before image output. As a result, our design is more conducive to the direct visual observation of the processed infrared image results by the human eye.

## 7. Conclusions

In this article, we propose a lightweight real-time infrared image processor based on FPGA. The processor utilizes the lightweight two-point correction algorithm introduced in this paper to remove NUs in the original infrared images. An approximate blind pixel detection algorithm is employed to calibrate blind pixels in the original infrared images, eliminating the need for extensive iterative calculations. The processor adopts a SWF blind pixel compensation algorithm based on SWF to handle blind pixels in the infrared images while preserving a significant amount of details from the original image. A lightweight histogram equalization algorithm is applied to enhance the processed infrared images for better human observation. The processor is implemented on a lightweight FPGA platform. At a working frequency of 50 MHz, the real-time processing speed of the infrared images can reach 30 FPS, meeting the requirements for real-time infrared image processing. Vivado synthesis and implementation results indicate that the processor’s maximum operating frequency can go up to 186 MHz. The simulation experiments and practical tests on the FPGA demonstrate that the proposed infrared image processor exhibits excellent processing results. It effectively eliminates NUs in infrared images, compensates for blind pixels while retaining image details, and significantly enhances image contrast through histogram equalization. Our final design exhibits considerable competitiveness among similar products in the same category. Due to commercial confidentiality, we have decided to release only a portion of the code for open-source purposes to assist colleagues in replicating the experiments (https://github.com/FrankZhang1994/Lightweight-and-Real-Time-Infrared-Image-Processor; accessed on 10 Februaru 2024). In the future, our research plan involves leveraging the developed infrared image processor to explore the recognition and calibration of various targets in processed infrared images.

## Figures and Tables

**Table 1:** Hardware resource consumption on XC7A100T-2 in the practical experiment.

Module	LUT	FF	BRAM	DSP48	IO
**Our design**	**10,894**	**9367**	**4**	**5**	**0**
Cortex-M0	4316	4892	8	3	26
TF card controller	1120	1536	5	0	12
DDR3 controller	7221	5630	0	0	75
Others components	1964	968	3	0	43
Overall	25,515 (40.24%)	22,393 (17.66%)	20 (15.19%)	8 (3.33%)	156 (54.74%)

**Table 2:** Implementation results and comparison with existing closely related designs.

Works	Func.	Frame Size	Device	Area	Op. Freq. (MHz)	Max. FPS	Power
[16]	TC ✓ BC ✗ HE ✗	500×500	AMD Zynq 7010 28 nm PL	3020 LUTs 4314 Registers 9.5 BRAM 43 DSPs	135	540	84 mW
[17]	TC ✓ BC ✓ HE ✗	256×256	Intel Stratix II 90 nm	19,596 ALUTs 20,118 Registers 225 KBytes RAM 148 9-bit DSPs	12.5 (Actual)	25 @12.5 MHz 180 @Max.	— *
[18]	TC ✓ BC ✗ HE ✗	640×480	AMD Spartan-6 45 nm	503 LUTs 259 Registers 15 DSPs	100	238	13 mW
[19]	TC ✓ BC ✓ HE ✗	640×480	16 nm FPGA ^†^	4293 LUTs 4261 Registers 5 BRAM 11 DSPs	300	—	2910 mW
[20]	TC ✓ BC ✓ HE ✗	320×240	AMD Zynq Ultrascale+ 14/16 nm	18,687 LUTs 32,007 Registers 26.5 BRAM 1 DSP	99 (Actual)	30	3710 mW
This work	TC ✓ BC ✓ HE ✓	640×480	AMD Artix-7 28 nm	10,894 LUTs 9367 Registers 4 BRAM 5 DSPs	50 (Actual) 186 (Max.)	30 @50 MHz 111 @Max.	1800 mW @50 MHz 6.97 W @186 MHz

^†^ No mention of the specific FPGA type. * Not mentioned. TC: two-point correction, BC: blind pixel compensation, HE: histogram equalization.

## Data Availability

All data can be provided upon reasonable request to the corresponding author.
